# Particle and Fibre Toxicology, a new journal to meet a real need

**DOI:** 10.1186/1743-8977-1-1

**Published:** 2004-12-03

**Authors:** Ken Donaldson, Paul Borm

**Affiliations:** 1 ELEGI Colt Laboratory Wilkie Building University of Edinburgh Medical School Teviot Place Edinburgh EH9 8AG Scotland UK; 2 Centre of Expertise in Life Sciences (CEL) Zuyd University PO Box 550 6400 AN HEERLEN Netherlands

## Abstract

This Editorial is to announce *Particle and Fibre Toxicology*, a new Open Access, peer-reviewed, online journal published by BioMed Central. The field of particle and fibre toxicology has a long and famous history stretching from Agricola and Paracelsus in the 15th and 16th century to the challenges of the 21st century-nanoparticles, nanotubes and particulate matter (PM10) to name just three. Throughout this time there has been no single journal dedicated to the toxicology of particles and fibres and this is finally corrected by the launch of *Particle and Fibre Toxicology*. The rationale for *Particle and Fibre Toxicology *rests on this need for a single multi-disciplinary journal that can cover all research relevant to particle and fibre toxicology, from Hygiene studies, through particle generation and characterisation, to animal, cell and human toxicology studies, dosimetry and modelling. The editorial also deals with the philosophy and practicalities of Open Access publishing, the journal's peer-review policy and conflict-of-interest. *Particle and Fibre Toxicology *is aimed at bringing together multi-disciplinary research findings towards a better understanding of how particles and fibres adversely affect the lungs and the body generally. We hope that the launch of the new journal will aid in the advance of this important discipline to the greater benefit of occupational and public health and invite scientists working in this key discipline to submit their research.

## Editorial

*Particle and Fibre Toxicology *is a new Open Access, peer-reviewed, online journal published by BioMed Central, which will publish articles pertaining to all aspects of the toxicological effects of particles and fibres. Particles and fibres are toxicologically important in many scenarios. There is exposure, leading to adverse health effects, during manufacture and use of industrial products, accidental exposure during disturbance of the earth's crust in mining and quarrying, accidental exposure from general anthropogenic sources in the environment, and exposure to organic particles containing biologically active compounds such as endotoxins and glucans. Traditionally the particles and fibres most intensively studied include, PM10, PM2.5, asbestos, synthetic vitreous fibres, metals, quartz (crystalline silica), and coalmine dust. There are, however, numerous other particle types under study. The diseases and conditions caused or affected by exposure to particles include cancer, fibrosis, asthma, chronic obstructive pulmonary disease (COPD) and cardiovascular disease. Recently there has been increasing interest in the extra-pulmonary effects of particles, such as transport of particles to the blood and brain and the consequences of such transport on neurological function, blood coagulation and cardiovascular health.

Recently a whole new class of particles, those in the nanoparticle size range, have come under intense scrutiny. Among those nanotubes and their toxicological effects are also receiving increased attention. Nanoparticles are increasingly used in 'Nanomedicine' for diagnostic, imaging and therapeutic purposes and these require special toxicological considerations [1].

## Overview of journal scope

*Particle and Fibre Toxicology *welcomes manuscripts dealing with the following toxicological aspects pertaining to particles and fibres: mechanistic studies, dosimetry, physicochemical factors in particle toxicity, animal studies, the role of size in particle toxicity, the role of composition in particle toxicity, cell studies, studies in acellular systems, biopersistence and durability studies, studies aimed at improving the dose metric, inhalation studies with real and surrogate particles, mathematical modelling, deposition and clearance studies, anatomical and histological studies, regulation and regulatory studies, pathology studies, risk assessment, translocation studies on the fate of particles, and all toxicological studies that throw light on how particles cause harmful effects in the lungs, liver, blood and nervous system. *Particle and Fibre Toxicology *especially welcomes manuscripts relating to the toxicological hazards and risks of nanoparticles and nanotubes especially when used for specific biomedical purposes. The journal will consider the following types of article: research, commentary, debate, hypothesis, methodology articles, reviews and short reports.

## Why is *Particle and Fibre Toxicology *needed?

The field of particle and fibre toxicology has a long and famous history. During the 15th and 16th centuries Agricola and Paracelsus were the first to document lung diseases caused by inhalation of dust and by the time of the Industrial Revolution in 18th and 19th century, the toll of ill-health from dusty trades was enormous, with industrial hygiene still in its infancy. By the 20th century the global epidemic of lung disease caused by asbestos and crystalline silica demonstrated that the toxicology of dusts and fibres was still 'a work in progress'. In the 21st century we face the old challenges plus new ones, such as environmental particulate air pollution, the new focus on the extra-pulmonary effects of particles and potential risks from nanoparticles arising from the nanotechnology industry. A journal dedicated to the toxicology of particles and fibres is a new and worthy addition to this long history.

Studies on topics related to particle and fibre toxicology translate into thousands of scientific articles each year but historically these have been scattered across numerous journals. The target toxicology journal is usually chosen on the basis of the endpoint under study, or if humans are exposed, occupational and environmental journals may be chosen. Those experienced in particle and fibre research appreciate the special expertise required to carry out and interpret toxicological studies because of potentially confusing factors such as absorption, aggregation, surface coating, dosimetry and overload. Such details are not always appreciated by non-specialists. *Particle and Fibre Toxicology *will provide a unique single outlet and focus of expertise in this multi-disciplinary subject. Those involved in research into particles and fibres include physicists, chemists, materials scientists, environmental scientists, hygienists, mechanistic toxicologists, inhalation toxicologists, cell biologists, medical specialists, epidemiologists and risk assessors. A single target journal specialising in particle and fibre toxicology will provide a focus for the diverse community employed in this global research enterprise. Focusing on the interface between particles/fibres and humans, *Particle and Fibre Toxicology *should aid in the advance of this important discipline to the ultimate improvement of occupational and public health.

## Peer review policy

Publication of research articles in the broad area of particle toxicology is dependent on: relevance to the aims of the journal; scientific validity and excellence; and coherence with the research area as a whole, as judged by two independent reviewers. An International Editorial Board oversee the peer review of manuscripts submitted to *Particle and Fibre Toxicology*. Peer review is anonymous. A recommendation of acceptance or rejection is at the discretion of the Editors based on the reviewers' recommendations and will be agreed in consultation with the reviewers. Articles will be published immediately upon acceptance (after peer review) and soon after they will be listed in PubMed and archived in PubMed Central.

## Open Access

*Particle and Fibre Toxicology*'*s *Open Access policy changes the way in which articles are published. First, all articles become freely and universally accessible online, and so an author's work can be read by anyone at no cost. Second, the authors hold copyright for their work and grant anyone the right to reproduce and disseminate the article, provided that it is correctly cited and no errors are introduced [2]. Third, a copy of the full text of each Open Access article is permanently archived in an online repository separate from the journal. *Particle and Fibre Toxicology*'s articles are archived in PubMed Central [3], the US National Library of Medicine's full-text repository of life science literature, and also in repositories at the University of Potsdam [4] in Germany, at INIST [5] in France and in e-Depot [6], the National Library of the Netherlands' digital archive of all electronic publications.

Open Access has four broad benefits for science and the general public. First, authors are assured that their work is disseminated to the widest possible audience, given that there are no barriers to access their work. This is accentuated by the authors being free to reproduce and distribute their work, for example by placing it on their institution's website. It has been suggested that free online articles are more highly cited because of their easier availability [7]. Second, the information available to researchers will not be limited by their library's budget, and the widespread availability of articles will enhance literature searching [8]. Third, the results of publicly funded research will be accessible to all taxpayers and not just those with access to a library with a subscription. As such, Open Access could help to increase public interest in, and support of, research. Note that this public accessibility may become a legal requirement in the USA if the proposed Public Access to Science Act is made law [9]. Fourth, a country's economy will not influence its scientists' ability to access articles because resource-poor countries (and institutions) will be able to read the same material as wealthier ones (although creating access to the internet is another matter [10]).

## Funding Open Access

6 months after the launch of *Particle and Fibre Toxicology *all articles accepted by the journal will be subject to an article processing charge of $525. The fee will be payable by the authors unless their institution is a BioMed Central member, but will be waived in cases of genuine hardship.

## Conclusion

The information revolution is a double-edged sword felt as keenly in science as anywhere else. We live in an age where health science is a multimillion pound/euro/dollar industry and its product, scientific articles, are produced in vast numbers. Scientists and regulators struggle to find this information and interpret it in ways that will benefit human health. This is particularly relevant in toxicology of particles and fibres because of its multidisciplinary nature. Open access publishing clearly helps us make our way through the forest of information by publishing rapidly and allowing easy access to research articles. Our new journal *Particle and Fibre Toxicology *should also help by acting as a focus for multidisciplinary research in this area. We therefore look on *Particle and Fibre Toxicology *as a significant opportunity to advance this important area of public and occupational health and invite scientists in this field to submit their work.

## Competing interests

The Editor-in-Chief and the Editorial Board of *Particle and Fibre Toxicology *have no financial incentive to accept manuscripts as they are not paid on the basis of the number of manuscripts accepted. In fact, they do not receive any financial remuneration for their involvement with the journal. We insist that decisions about a manuscript are based on the quality of the work, not on whether the authors' can pay the article-processing charge. Authors will be asked to declare any competing interests that they have.
